# Life-threatening bleeding after pelvic lymphadenectomy for cervical cancer: endovascular management of ruptured false aneurysm of the external iliac artery

**DOI:** 10.1186/1477-7819-10-149

**Published:** 2012-07-16

**Authors:** Enzo Ricciardi, Giampaolo Di Martino, Paolo Maniglio, Mauro Schimberni, Antonio Frega, Marina Jakimovska, Borut Kobal, Massimo Moscarini

**Affiliations:** 1 Department of Obstetrics and Gynecology, Sapienza University of Rome, Sant'Andrea Hospital, via di Grottarossa, 1035-1039, Roma 00189, Italy; 2 Ginekoloska Klinika, University Medical Center, Ljubljana, Slovenia

**Keywords:** Cervical cancer, External iliac artery pseudo-aneurysm, Fertility-sparing procedure, Laparoscopy, Lymphadenectomy complication, Massive bleeding

## Abstract

Late rupture of external iliac artery pseudo-aneurysm is an uncommon complication in patients who undergo extensive gynecologic radical surgeries. A 28-year-old woman with stage IB cervical cancer underwent pelvic lymphadenectomy and extrafascial trachelectomy. Two months after surgery, massive bleeding from ruptured pseudo-aneurysm of the external iliac artery occurred. Endovascular management with covered stent placement was feasible and safe to stop bleeding.

## Background

Iatrogenic pelvic pseudo-aneurysms following gynecologic or urologic procedures have been rarely reported in the past. We present a patient who had a previous surgery for cervical cancer who had sudden life-threatening bleeding due to a ruptured pseudo-aneurysm of the right external iliac artery. The placement of a covered stent was successful to stop the bleeding and assured a safe outcome for the patient.

## Case presentation

A 28-year-old Caucasian woman, gravida 0 para 0, with regular menses, was diagnosed with “undifferentiated squamous carcinoma of the cervix” after a biopsy was performed.

MRI of the abdomen confirmed the presence of an expansive exocervical formation located posteriorly with a maximum diameter of 2 cm. MRI staging: Figo stage IB.

Metastatic work-up, including computed tomography of thorax and abdomen, was negative.

The procedure included bilateral radical pelvic lymphadenectomy and extrafascial trachelectomy. Body mass index of 39 was not considered a contra-indication for laparoscopy, which was successfully performed in order to remove pelvic lymph nodes. Total operative time was 3½ hours. Blood loss was 150 mL. Histological examination revealed a poorly differentiated squamous cell carcinoma of the cervix. Lymph-vascular space invasion (LVSI) was negative. Tumor stage was pT1B, G3, pN0 (pelvic nodes 0/35), IB (Ajcc 2010).

Early postoperative course was uneventful. The patient was discharged after three days.

**Figure 1:**
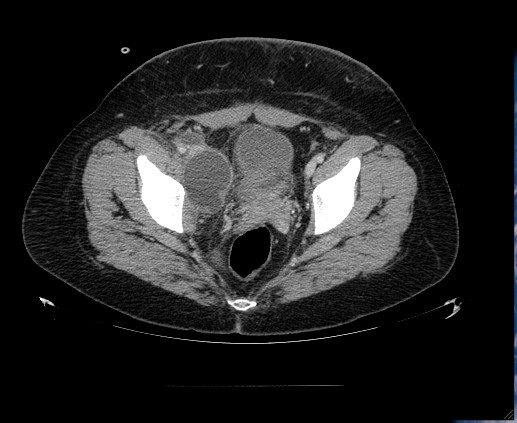
Fluid-filled sac (cm 15 × 6 × 5) on the right psoas major muscle.

One month later, the patient presented with massive lymphedema of the right inferior limb. Imaging showed deep femoral vein thrombosis; anticoagulants were administered. CT scans of the abdomen showed a fluid-filled pelvic mass (measuring 15 × 6 × 5 cm) lying on the psoas major (Figure 1).

A pelvic drain was placed into the mass under fluoroscopic guide.

Nevertheless, the patient was soon readmitted for pelvic pain associated with the signs of a thrombophlebitis of the right thigh.

Imaging demonstrated that a lymphocele kept dislocating the iliac vein and compressed the iliac artery posteriorly. Anticoagulants were administered at a higher dose. The pelvic drain was replaced and the patient was discharged in good condition after 20 days hospitalization.

**Figure 2:**
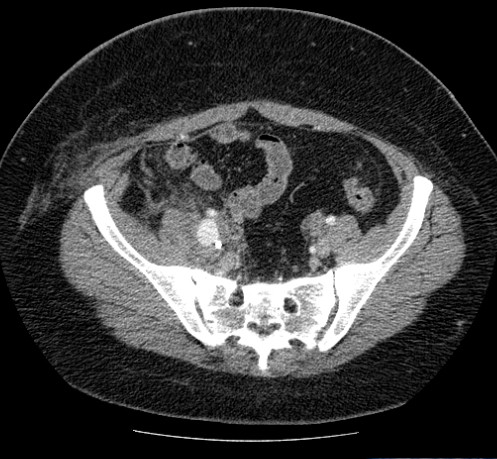
Active bleeding in the iliopsoas compartment.

Fifteen days later the patient presented spontaneously at the emergency department after noticing blood traces in the pelvic drain. CT scan with contrast demonstrated active bleeding at the psoas major area (Figure 2).

**Figure 3:**
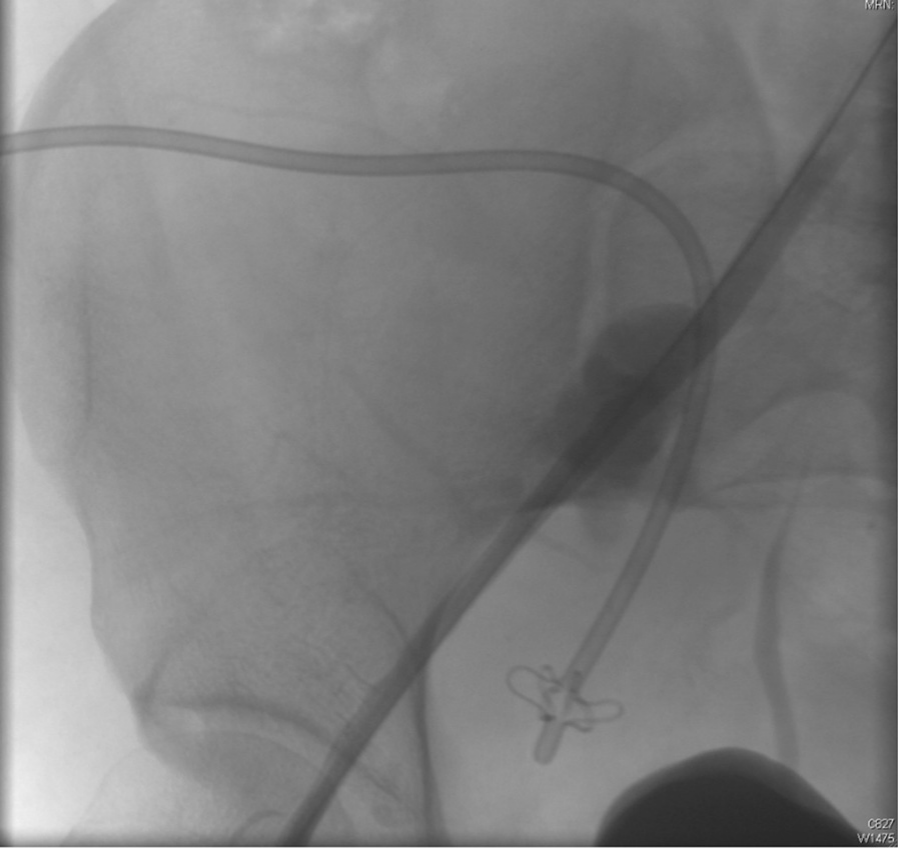
False aneurysm of the external iliac artery.

**Figure 4:**
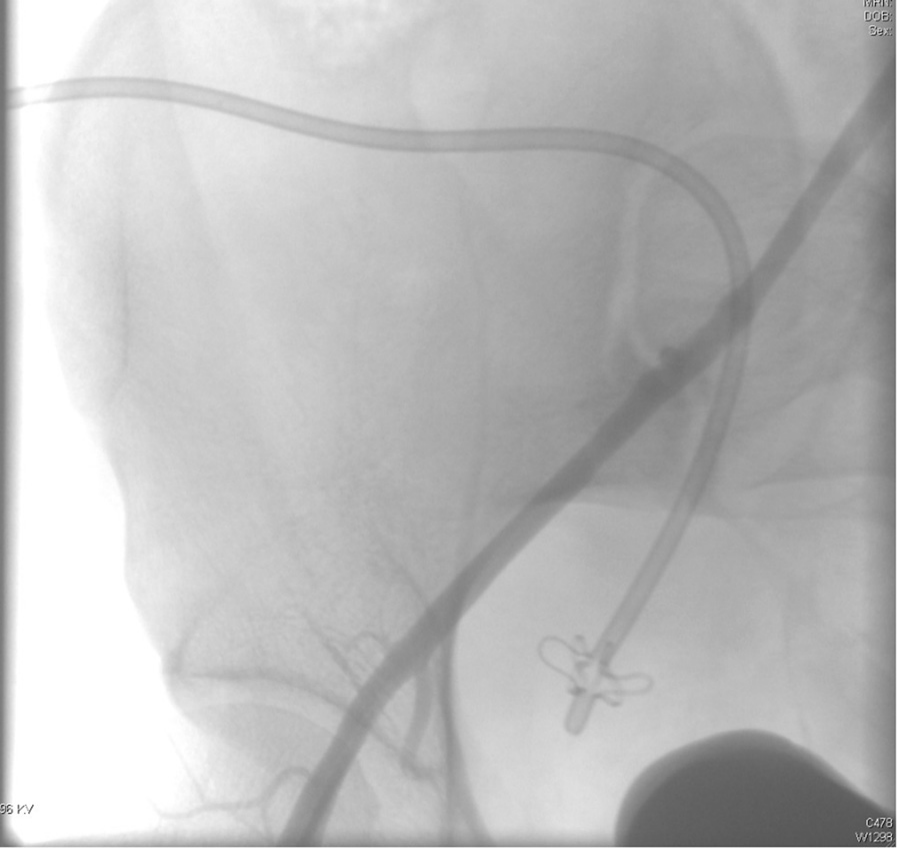
Ruptured wall of the artery.

The interventional radiologist was, therefore, alerted and emergency surgery was planned. Before entering the operating theater, the patient suffered massive blood loss from the pelvic drain (the sac filled 800 cc in five minutes). Arteriography demonstrated a ruptured pseudo-aneurysm of the external iliac artery on the right side (Figure 3). A lesion in the vessel wall was clearly seen in the posterior aspect of the artery (Figure 4). A covered stent (Gore® Viabahn® W. L. Gore & Associates, Inc. Medical Products Division Arizona USA), measuring 7 mm in diameter and 50 mm long, was placed after percutaneous transluminal angioplasty (PTA) with a balloon catheter (Invatec Admiral Xtreme™ (INVATEC Inc. Bethlehem, Pennsilvania USA) Postoperatively the patient recovered well and had no further complications after four months of follow-up.

## Discussion

Pseudo-aneurysms are consequences of arterial wall damage. The hematoma resulting becomes confined in the adjacent tissues by fascial planes [1]. Common causes are trauma, tumor, infection, vasculitis and inflammation (even caused by radiation therapy), atherosclerosis, infarction and various iatrogenic complications, such as the ones from surgery and angiography [2]. It is not clear whether arterial injury related to surgery might develop but it is well known that the complication of a pseudo-aneurysm can appear weeks or months after surgery [3]. Our case is the first report to our knowledge of a late false aneurysm rupture after cervical cancer surgery, being apparently related to the sole procedure. There are few similar cases previously reported [1,2,4]. Most of them have been associated with radiation therapy delivered before the onset of the complication [2,4]. In our case, the formation of the pseudo-aneurysm might be surgery-related as no other apparent causes were present.

Deep venous thrombosis of the femoral vein was likely to be caused by compression of the iliac vein as it appeared displaced by the mass.

Rupture of false aneurysms with massive blood loss is a serious life-threatening condition and requires immediate surgical correction of the arterial wall defect. An endovascular approach with covered stent placement appears to be successful and it should be the first choice in our opinion in these patients. The clearest advantage of this former approach, when compared to open surgery, is a much quicker control of the bleeding, which is paramount in these extremely urgent situations.

Our patient was young and without appearance of vascular disease so we do not have concerns about long-term patency. The consolidated experience with covered stents in older patients with vascular disease shows that both proximal and distal parts of the stent have to land in a healthy portion of the vessel as it was in our case. This is key to prevention of stent re-stenosis.

The choice of a stent like Viabahn® was made on our center experience in both urgent and elective settings. The primary patency rate for this stent at 12, 24, 36, and 48 months was 72 %, 63 %, 63 % and 59 %, respectively [5]. Other stents are also available, such as Fluency® by BARD® medical, Covington, Georgia but in our case we believe there was no advantage in using this latter type.

One difference between stents is that Viabahn® is covered with one inner layer of expanded polytetrafluoroethylene (ePTFE) while Fluency® is covered with two layers (outer and inner) of ePTFE. Two layers of Gore-Tex® W. L. Gore & Associates, Inc. Medical Products Division Arizona USA (ePTFE) allow better resistance to external muscular compression and repetitive stress to the artery when stent is placed in peripheral vessels (for example; superficial femoral artery (SFA) in the Hunter’s canal).

Muscles or other physiological sources of potential stress or compression do not surround the external iliac artery. This makes us think that the stent choice was appropriate.

## Conclusions

We believe that our case and its management are important and to be taken into consideration for the severity of the condition any time a lymphadenectomy is performed, even without any other apparent risk factor for vascular damage.

## Consent

Written informed consent was obtained from the patient for publication of this Case report and any accompanying images. A copy of the written consent is available for review by the Editor-in-Chief of this journal.

## Abbreviations

ePTFE: Expanded polytetrafluoroethylene; LVSI: Lymph-vascular space invasion; PTA: Percutaneous transluminal angioplasty; SFA: Superficial femoral artery.

## Competing interests

The authors declare that there are no conflicts of interest.

## Authors’ contribution

ER conceived of the study, participated in its design and coordination, and drafted the manuscript. GDM participated in the design of the study and manuscript revisions. PM participated in collecting data, and read and corrected the manuscript. MS participated in the design of the study and in manuscript revisions. AF gave intellectual input and corrected the manuscript. MJ participated in collecting data, and read and corrected the manuscript. BK participated in the design of the study and manuscript revisions. MM helped in editing, reading and correcting the manuscript. All authors read and approved the final manuscript.
